# A High Degree of LINE-1 Hypomethylation Is a Unique Feature of Early-Onset Colorectal Cancer

**DOI:** 10.1371/journal.pone.0045357

**Published:** 2012-09-25

**Authors:** Marina Antelo, Francesc Balaguer, Jinru Shia, Yan Shen, Keun Hur, Leticia Moreira, Miriam Cuatrecasas, Luis Bujanda, Maria Dolores Giraldez, Masanobu Takahashi, Ana Cabanne, Mario Edmundo Barugel, Mildred Arnold, Enrique Luis Roca, Montserrat Andreu, Sergi Castellvi-Bel, Xavier Llor, Rodrigo Jover, Antoni Castells, C. Richard Boland, Ajay Goel

**Affiliations:** 1 Oncology and Pathology Sections, Hospital of Gastroenterology “Dr. C. B. Udaondo”, Buenos Aires, Argentina; 2 Department of Internal Medicine, Division of Gastroenterology, Charles A. Sammons Cancer Center and Baylor Research Institute, Baylor University Medical Center, Dallas, Texas, United States of America; 3 Department of Gastroenterology, Hospital Clínic, Centro de Investigación Biomédica en Red de Enfermedades Hepáticas y Digestivas (CIBERehd), Institut d’Investigacions Biomediques August Pi i Sunyer (IDIBAPS), University of Barcelona, Barcelona, Catalonia, Spain; 4 Department of Pathology, Memorial Sloan Kettering Cancer Center, New York, New York, United States of America; 5 Department of Gastroenterology, CIBERehd, University of Country Basque, Donostia Hospital, San Sebastián, Spain; 6 Deparment of Gastroenterology, Hospital del Mar, Barcelona, Spain; 7 Department of Medicine and Cancer Center, University of Illinois at Chicago, Chicago, Illinois, United States of America; 8 Gastroenterology Unit, Hospital General Universitario, Alicante, Spain; The Chinese University of Hong Kong, Hong Kong

## Abstract

**Objective:**

Early-onset colorectal cancer (CRC) represents a clinically distinct form of CRC that is often associated with a poor prognosis. Methylation levels of genomic repeats such as LINE-1 elements have been recognized as independent factors for increased cancer-related mortality. The methylation status of LINE-1 elements in early-onset CRC has not been analyzed previously.

**Design:**

We analyzed 343 CRC tissues and 32 normal colonic mucosa samples, including 2 independent cohorts of CRC diagnosed ≤50 years old (n = 188), a group of sporadic CRC >50 years (MSS n = 89; MSI n = 46), and a group of Lynch syndrome CRCs (n = 20). Tumor mismatch repair protein expression, microsatellite instability status, LINE-1 and *MLH1* methylation, somatic *BRAF* V600E mutation, and germline *MUTYH* mutations were evaluated.

**Results:**

Mean LINE-1 methylation levels (±SD) in the five study groups were early-onset CRC, 56.6% (8.6); sporadic MSI, 67.1% (5.5); sporadic MSS, 65.1% (6.3); Lynch syndrome, 66.3% (4.5) and normal mucosa, 76.5% (1.5). Early-onset CRC had significantly lower LINE-1 methylation than any other group (p<0.0001). Compared to patients with <65% LINE-1 methylation in tumors, those with ≥65% LINE-1 methylation had significantly better overall survival (p = 0.026, log rank test).

**Conclusions:**

LINE-1 hypomethylation constitutes a potentially important feature of early-onset CRC, and suggests a distinct molecular subtype. Further studies are needed to assess the potential of LINE-1 methylation status as a prognostic biomarker for young people with CRC.

## Introduction

Colorectal cancer (CRC) is an important public health problem and represents the second most frequent cancer and the second greatest cause cancer-related mortality in most of the developed world. Each year, one million people develop CRC, and 40–50% of them will die within 5 years of diagnosis [1]. CRCs are highly heterogeneous both histopathologically, and at the molecular and genetic level. It appears that the biology and response to therapies is equally diverse. Understanding the molecular mechanisms of colorectal carcinogenesis is essential for the development of new strategies for prevention, diagnosis, treatment and prognosis. Although CRC has been a major focus of attention for basic and clinical research during the last 25 years, we still lack robust biomarkers that can be used for diagnosis and treatment of CRC.

The peak incidence of CRC is between 60–70 years old; however up to 10% of all cases occur before age 50. Moreover, recent epidemiological studies suggest that the incidence of early-onset CRC is increasing, representing an important clinical challenge [2]. Early-onset CRC often presents with advanced stage tumors, which contributes to a higher rate of mortality [3]. Since young people are not included in CRC screening programs, there is an urgent need to understand the biology of early-onset tumors, which could facilitate earlier detection and treatment of these cancers.

Although early-onset CRC raises the possibility of a hereditary risk factor, the known non-polyposis hereditary CRC syndromes (Lynch Syndrome and *MUTYH*-associated CRC) represents no more than 15–20% of cases in this group [4], [5], [6]. Lynch Syndrome accounts for about 3% of all CRC cases, and is caused by germline mutations of the DNA mismatch repair (MMR) genes (*MLH1, MSH2, MSH6* and *PMS2*) [7]. It is characterized by early-onset cancers arising in the colorectum and other organs, and there are currently several strategies and algorithms to predict the presence of a germline mutation in one of the MMR genes [8], [9], [10], [11]. Biallelic mutations in the *MUTYH* gene (a member of the base excision repair system) accounts for<1% of all CRC, and usually causes an attenuated form of polyposis, although 30% of these patients can manifest as a non-polyposis CRC [12].Identifying individuals with germline mutations that predispose to CRC has significant implications for the clinical management of affected individuals and for their relatives.

The remaining 75–80% of early-onset CRC represents another group in which the genetic etiology has not yet been discovered. In contrast to CRC on older individuals, early-onset CRC is often characterized by more advanced stage, distal location (especially in rectum), mucinous and poorly differentiated tumors with signet ring cells, and a poorer prognosis [4], [13], [14]. The majority of these cancers do not show microsatellite instability (MSI), but rather are microsatellite stable (MSS). The molecular basis for the biological and behavioral differences in early-onset CRC is unclear.

Genome-wide DNA hypomethylation is a frequent epigenetic alteration that is an early event in CRC and has been associated with the activation of certain proto-oncogenes (i.e. MET) [15] and the presence of chromosomal instability [16], [17]. Global DNA hypomethylation can be measured indirectly by assessing the methylation status of long interspersed nucleotide element-1 (LINE-1) repeat sequences [18]. The pyrosequencing assay for LINE-1 methylation has been found to be quantitative, robust and reproducible [19]. The degree of LINE-1 hypomethylation has been recognized as an independent factor for increased cancer-related mortality and overall mortality in CRC patients [20]. Although it has been suggested that LINE-1 hypomethylation is associated with CRC in younger patients [21], the specific association between methylation status of LINE-1 elements and early-onset CRC has not been further analyzed.

The aim of this study was to characterize the clinical, histological, and molecular features of a large cohort of early-onset CRCs in the context of the methylation status of LINE-1 elements. Our results indicate that LINE-1 hypomethylation in these tumors constitutes a unique and specific feature, which is suggestive of a distinct molecular subtype in these colorectal neoplasms. Our findings suggest that LINE-1 methylation status could be used as a prognostic biomarker for young people with CRC.

## Patients and Methods

### Ethics Statement

The study was approved by the Institutional Review Boards (IRB) of each participating center, including Hospital of Gastroenterology “Dr. C. B. Udaondo”, Buenos Aires, Argentina, Baylor University Medical Center, Dallas, TX and the Epicolon consortium. A written informed consent was obtained from all patients.

### Patients

We analyzed 343 CRCs from different clinic-pathological groups, and 32 normal colonic mucosa samples. We included a cohort of 118 retrospectively recruited CRC patients ≤50 years old seen at the Oncology Section of the Argentine Public Hospital of Gastroenterology between 1993 and 2009. Patients with colorectal polyposis or inflammatory bowel disease were excluded. Demographic and clinic-pathological features were collected from each patient’s medical history, and family history of cancer in first and second degree relatives was obtained by personal interview. The median follow-up time was 39 months (range, 1.5–195 months). For the LINE-1 methylation analyses, as a validation group, we included a previously described cohort of 70 patients with CRC diagnosed ≤50 years old treated at two Spanish centers (Hospital Clinic of Barcelona and Hospital of Donostia) between 1995–2007 [4]. We also included a population-based cohort of sporadic CRCs>50 years recruited in Spain (Epicolon I study) [9] categorized by the presence of MSI (“sporadic MSI” due to somatic *MLH1* promoter hypermethylation [n = 46], and “sporadic MSS” [n = 89]); and a group of Lynch syndrome CRCs recruited at Baylor University Medical Center at Dallas (n = 20). We histologically analyzed normal colonic mucosa from 32 individuals undergoing colonic surgery for reasons other than cancer (i.e. diverticulosis) recruited in the Hospital Clinic of Barcelona, Spain.

### DNA Isolation

Genomic DNA from each patient were extracted from formalin-fixed paraffin-embedded (FFPE) microdissected tumor tissues using the QiaAmp Tissue Kit (Qiagen, Courtaboeuf, France) according to the manufacturers’ instructions. Peripheral blood DNA was extracted using the QiaAmpDNA blood Mini Kit (Qiagen, Courtaboeuf, France). For rectal tumors in which chemo-radiotherapy was administered, pre-treatment tissue samples were analyzed.

### Tumor Mismatch Repair Protein Expression

One block of FFPE tumor tissue was selected per case and immunostaining was performed using standard protocols. The following mouse monoclonal antibodies were used: anti-MLH1 (clone G168-728, diluted 1∶250, PharMingen, San Diego, CA), anti-MSH2 (clone FE11, diluted 1∶50, Oncogene ResearchProducts, Cambridge, MA), anti-MSH6(clone GRBP.P1/2.D4, diluted 1∶200; Serotec Inc, Raleigh, NC) and anti-PMS2 (clone A16-4, diluted 1∶200, BD PharMingen, San Diego, CA). A tumor was deemed negative for protein expression only if the neoplastic epithelium lacked nuclear staining, while non-neoplastic epithelial or stromal cells retained normal expression of that protein.

### Tumor Microsatellite Instability Analysis

MSI analysis was carried out using five mononucleotide repeat microsatellite targets (BAT-25, BAT-26, NR-21, NR-24 and NR-27) in a pentaplex PCR system. Primer sequences have been described previously [22]. Tumors with instability at ≥3 these markers were classified as microsatellite unstable (MSI) and those showing instability at ≤2 markers as microsatellite stable (MSS). The researchers scoring immunostaining were blinded to the MSI results, and vice versa.

### Methylation Analyses

DNA was modified with sodium-bisulfite using the EZ Methylation Gold Kit (Zymo Research, Orange, CA). Methylation of LINE-1 sequences and the promoter of *MLH1* was analyzed by quantitative bisulfite pyrosequencing as described previously [23]. Primers are detailed in **Table S1**.

### Germline MUTYH Gene Mutation Analysis

All patients were screened for the two most prevalent *MUTYH* mutations in Caucasian populations (p.G393D and p.Y176C) by pyrosequencing. Primers are detailed in **Table S1**. In heterozygotes for any of these mutations, the coding region and exon-intron boundaries of the *MUTYH* gene were screened by SSCP with sequencing of abnormal band shifts, as described previously [12].

### Somatic BRAF V600Emutation Analysis

The *BRAF* V600E mutational analysis was performed by pyrosequencing. The PCR and sequencing primers are detailed in **Table S1**.

### Statistical Analysis

Quantitative variables were compared using Student’s test. Qualitative variables were compared using either the Chi Square or the Fisher’s test when appropriate. For associations with MMR deficiency and LINE-1 methylation (treated as a binary variable) a multivariate analysis using a stepwise backward logistic regression procedure was performed to assess the independent associations. The Mann Whitney test was used to compare LINE-1 values. Overall survival associated with clinic-pathological and molecular variables (tumor stage, MMR deficiency, tumor location, family history of CRC, tumor differentiation, mucinous component, tumor infiltrating lymphocytes and LINE-1 methylation) were calculated by the Kaplan–Meier method (log rank test). A two sided p-value of <0.05 was regarded as significant. SPSS v17 software was used for statistical analysis.

## Results

### Patient’s Characteristics

We recruited 118 patients with early-onset CRC. Clinico-pathological features are shown in **Table 1**. The mean age at diagnosis was 37 years (standard deviation (SD), 8.25), and 61 (51.7%) patients were female. In 34 (28.8%) the tumor was proximal to the splenic flexure, 35 (29.6%) were in the distal colon, and 49 (41.6%) were in the rectum. At presentation, 22 (18.6%) patients had 1–10 synchronous adenomas; 18 presented with 1–5 adenomas and 4 patients had 6–10 adenomas. Three cases (2.5%) had a synchronous tumor (2 CRC and 1 neuroendocrine tumor in the appendix), and 5 (4.2%) developed a metachronous tumor during follow-up (4 CRC and 1 urothelial carcinoma). The majority of cases (77; 65.3%) were diagnosed at advanced stages (III-IV). Poorly differentiated tumors were seen in 15 (13.1%) patients, 41 (34.7%) had mucinous features and 65 (55%) had pathological features suggestive of the MSI phenotype, with one or more of the following: signet ring cells, Crohn’s-like lymphocytic reaction, tumor infiltrating lymphocytes, medullary growth pattern, or anaplastic features. More than 85% (n = 100) of the patients had experienced abdominal pain prior to diagnosis, 83 (70%) presented with an alteration in bowel habits, 71 (60%) had rectal bleeding and weight loss, 34 (29%) had iron deficiency anemia, 18 (15.5%) presented with bowel obstruction, and 6 (5%) with perforation. The average delay between initial symptoms and CRC diagnosis was 6.5±5 months. Fifteen patients (12.7%) had a family history of CRC or another Lynch syndrome-associated neoplasm in first or second-degree relatives. Three patients met Amsterdam I criteria, 3 patients met Amsterdam II criteria, 4 patients had one first degree relative with CRC, 3 patients had two or more second degree relatives with CRC, and 2 patients had one second degree relative with CRC.

**Table 1:** Clinical, pathological and molecular features of patients with mismatch repair deficiency.

Clinical, pathological or molecular feature	Cohort N = 118	MMR deficient^1^N = 27 (22.9%)	MMR proficient^2^N = 91 (77.1%)	p-value
Age at diagnosis, mean (standard deviation)	37 (8.25)	35 (10.06)	38 (7.55)	0.23
Age range	(29–45)	(25–45)	(30–45)	
Sex, n (%)				
Female	61 (51.7)	13 (48.1)	48 (52.7)	0.67
Male	57 (48.3)	14 (51.9)	43 (47.3)	
Tumor location, n (%)				
Rectum	49 (41.5)	6 (22.2)	43 (47.3)	0.0001
Distal to splenic flexure	35 (29.7)	5 (18.5)	30 (33)	
Proximal to splenic flexure	34 (28.8)	16 (59.3)	18 (19.8)	
Synchronous or metachronous CRC, n (%)				
Yes	6 (5.1)	3 (11.1)	3 (3.3)	0.132
No	112 (94.9)	24 (88.9)	96.7)	
Synchronous adenomas, n (%)				
0	81 (68.6)	18 (66.7)	63 (69.2)	0.589
1–5	18 (15.2)	6 (22.2)	12 (13.2)	
6–10	4 (3.4)	1 (3.7)	3 (3.3)	
Incomplete colonoscopy	15 (12.8)	2 (7.4)	13 (14.3)	
Synchronous hyperplastic polyps, n (%)				
0	95 (80.5)	24 (86.2)	71 (78.0)	0.644
1–5	7 (6)	1 (6.9)	6 (6.6)	
6–10	1 (0.70)	0 (0)	1 (1.1)	
Incomplete colonoscopy	15 (12.8)	2 (6.9)	13 (14.3)	
Family history of CRC or other Lynch syndrome associated neoplasia^3^, n (%)				
Yes	15 (12.7)	5 (18.5)	10 (11.0)	0.30
No	103 (87.3)	22 (81.5)	81 (89.0)	
TNM tumor stage, n (%)				
I–II	41 (34.7)	14 (51.9)	27 (29.7)	0.03
III–IV	77 (65.3)	13 (48.1)	64 (70.3)	
Tumor differentiation, n (%)				
Well or moderate	100 (86.9)	24 (88.9)	76 (86.3)	1
Poor	15 (13.1)	3 (11.1)	12 (13.7)	
Mucinous component, n (%)				
>50%	41 (34.7)	17 (63)	24 (26.4)	0.0001
<50%	77 (65.3)	10 (37)	67 (73.6)	
Tumor infiltrating lymphocytes, n (%)				
Yes	26 (22.8)	16 (59.3)	10 (11.5)	0.0001
No	88 (77.2)	11 (40.7)	77 (88.5)	
Medullary growth pattern, n (%)				
Yes	11 (9.4)	3 (11.1)	8 (8.9)	0.714
No	106 (90.6)	24 (88.9)	82 (91.1)	
Tumors withCrohńs reaction, n (%)				
Yes	12 (10.6)	5 (18.5)	7 (8.1)	0.154
No	101 (89.4)	22 (81.5)	79 (91.9)	
Pathology suggestive of MSI^4^, n (%)				
Yes	65 (55)	22 (81.5)	43 (47.2)	0.002
No	53 (45)	5 (18.5)	48 (52.8)	
Somatic *BRAF* mutations, n (%)				
Wild-type	108 (96.4)	25 (96.2)	83 (96.5)	1
Mutated	4 (3.6)	1 (3.8)	3 (3.5)	
LINE-1 methylation, mean (standard deviation)	59.97 (6.57)	61.26 (6.13)	59.7 (6.68)	0.244
Progression/recurrence				
Yes	46 (39)	6 (22.2)	40 (44)	0.042
No	72 (61)	21 (77.8)	51 (56)	
Three-year overall survival	84.7%%	96.3%	83.5%	0.115

1 MSI-H and/or loss of expression of MMR proteins by immunohistochemistry.

2 MSS and normal expression of MMR proteins by immunohistochemistry.

3 Including first and second degree relatives; Lynch syndrome-associated neoplasia includes: endometrium, stomach, ovaries, urinary tract, small intestine, pancreas, bile ducts, brain or sebaceous glands.

4 Signet ring cells and/or Crohn’s-like lymphocytic reaction and/or tumor infiltrating lymphocytes and/or medullary growth pattern and/or anaplastic tumor.

### Germline MUTYH Gene Mutation Analysis

Biallelic MUTYH mutations were found in 1/91 MMR-proficient cases (1.1%)(****). This single case was a 29-year-old patient with a stage III rectal cancer and 2 synchronous adenomas. Two siblings of this patient had a history of attenuated polyposis and CRC (one presented with 30 adenomas and the other with 8 adenomas and an *in situ* carcinoma in the cecum); in both siblings total colectomies had been performed. Finally, we identified two p.G393D heterozygous patients that had no specific clinico-pathological features (**Table S2**).

### Mismatch Repair Deficiency Analysis

MMR deficiency was evaluated by MSI analysis and immunohistochemistry, and was defined by the presence of MSI in a tumor, and/or loss of expression in any of the MMR proteins. Twenty seven (22.9%) tumors were classified as MMR deficient, and 25 of these showed loss of protein expression (8 for MLH1/PMS2, 1 for isolated MLH1, 4 for isolated PMS2, 11 for MSH2/MSH6, and 1 for isolated MSH6). Clinico-pathological features of patients with MMR deficiency are summarized in **Table 2**.

**Table 2:** Clinicopathological and molecular features of patients with MMR deficiency.

Case	Age/Sex	Location	Stage	MSI	Immunohistochemistry*				Other tumors	Family history^2^	*BRAF status*	*MLH1*methylation	LINE-1 methylation (%)
					MLH1	PMS2	MSH2	MSH6				*(%)*	
2ARG	46 F	Ascending	II	MSI	P	P	L	L	Colon (46)	No	wt		53
63ARG	12 M	Rectum	IV	MSI	P	P	L	L	No	No	wt		53
90ARG	19 M	Caecum	IV	MSI	P	P	L	L	No	No	wt		59
24ARG	34 M	Hepatic flexure	I	MSI	P	P	L	L	No	No	wt		69
23ARG	30 F	Sigmoid	III	MSI	P	P	L	L	No	No	ND		71
71ARG	38 F	Rectum	II	MSI	P	P	L	L	No	Pancreas (mother, 52); Colon (uncle, 55); Colon (cousin, 44)	wt		60
16ARG	52 F	Caecum	III	MSI	P	P	L	L	No	No	wt		63
97ARG	54 F	Ascending	II	MSI	P	P	L	L	No	No	wt		59
84ARG	56 M	Rectum	I	MSI	P	P	L	L	Colon (40)	Colon (sister, 37 and 49); Colon (father, 37)	wt		66
62ARG	57 F	Rectum	III	MSI	P	P	L	L	No	No	wt		49
76ARG	53 M	Caecum	II	MSI	P	P	L	L	No	No	wt		51
77ARG	59 F	Ascending	III	MSS	P	P	P	L	No	Colon (grandmother, 65)	wt		62
19ARG	37 F	Caecum	II	MSI	L	L	P	P	No	No	wt	2	66
101ARG	43 F	Descending	II	MSI	L	L	P	P	No	No	wt	27	64
46ARG	42 M	Caecum	II	MSI	L	L	P	P	No	No	wt	25	59
93ARG	33 M	Splenic flexure	II	MSI	L	L	P	P	No	No	wt	1	61
49ARG	43 M	Caecum	II	MSI	L	L	P	P	No	No	wt	26	63
37ARG	49 F	Caecum	III	MSI	L	L	P	P	No	No	mutated	88	64
38ARG	50 M	Descending	III	MSI	L	L	P	P	No	No	wt	2	66
115ARG	55 F	Rectum	II	MSI	L	L	P	P	No	No	wt	51	64
21ARG	15 F	Caecum	III	MSS	P	L	P	P	No	No	wt		71
113ARG	36 M	Descending	III	MSI	P	L	P	P	No	No	wt		47
18ARG	58 M	Hepatic flexure	II	MSI	P	L	P	P	No	No	wt		62
79ARG	51 M	Transverse	III	MSI	P	L	P	P	No	Colon (aunt, 76)	wt		61
108ARG	41 M	Ascending	II	MSI	L	P	P	P	No	No	wt	1	65
112ARG	28 F	Rectum	III	MSI	P	P	P	P	No	No	wt		63
82ARG	26 M	Caecum	III	MSI	P	P	P	P	No	Colon (mother, 52); Colon (aunt, 46)	wt		63

MMR: mismatch repair; MSI: microsatellite instability; MSS: microsatellite stability; ND: not determined; wt: wild-type; P: present; L: loss of expression.

1 Solid cells indicate loss of protein expression,

^2^Affected relative and age at diagnosis are indicated between parentheses.

Nearly all cases of MSI had loss of protein expression; two cases with MSI retained normal expression of all four proteins. Likewise, 1 case with loss of MSH6 expression and one case with loss of PMS2 were MSS. The last patient was a 24 year-old woman who had CRC at age 15, a urothelial carcinoma at age 23, a metachronous CRC at age 24, and finally, a mediastinal B-cell lymphoma. Her CRC specimen showed loss of expression of PMS2 in tumor cells and in normal colonic surrounding tissue, leading to a presumptive diagnosis of constitutional MMR-deficiency syndrome due to bi-allelic PMS2 mutations.

As shown in **Table 1**, compared to MMR-proficient tumors, MMR-deficient tumors were more likely to be located in the proximal colon (59.3% *vs*. 19.8%, p = 0.0001), to be mucinous (63% *vs*. 26.4%, p = 0.0001), to have tumor infiltrating lymphocytes (59.3% *vs.* 11.5%, p = 0.0001), and to have MSI-suggestive pathology (81.5% *vs*. 47.2%, p = 0.002). MMR deficient tumors were also more likely to be diagnosed at a lower stage (stages I–II: 51.9% *vs*. 29.7%, p = 0.03), and to have less tumor recurrence or progression (22.2% *vs*. 44%, p = 0.042). Multivariate analysis showed that independent variables associated with MMR deficiency were: proximal location (OR = 3.86 [95%CI: 1.32–11.32]; p = 0.013), mucinous features (OR = 3.38 [95%CI: 1.16–9.84]; p = 0.025) and presence of tumor infiltrating lymphocytes (OR = 8.8 [95%CI: 2.93–26.31]; p = 0.0001). Although there was no difference in the age of CRC diagnosis between the 2 groups, the chance of having a MMR-deficient tumor was greater among younger patients (12–30 years: 8/27, 29.6%; 31–40 years: 9/45, 20%, 41–50 years: 10/46, 21.7%).

Somatic *BRAF* mutation was present in one MMR-deficient tumor (**Table 2**). This case was a 49-year-old male with an MSI tumor in the cecum that showed loss of MLH1 and PMS2 protein expression. This case showed high degree of *MLH1* promoter methylation (88%) and was therefore likely associated with CpG island methylator phenotype (CIMP) [24]. In the rest of the*MLH1*-deficient tumors, presumably carriers of *MLH1* germline mutations, 4 showed very low levels of methylation (range, 1–2%), and the other 4 showed intermediate levels (range, 25–51%).

### LINE-1 Methylation Analysis

We used the quantitative bisulfite pyrosequencing method to determine the methylation status of LINE-1 repetitive sequences in the CRCs. The average methylation in the CRCs was 59.97% (standard deviation, 6.57), which followed a normal distribution (**Figure S1**). Clinico-pathological features associated with LINE-1 methylation are shown in **Table 3**. A significant difference in LINE-1 methylation status was found according to tumor location, with lower levels of methylation in distal compared with proximal tumors (59.02% *vs.* 62.3%, p = 0.015). In addition, a trend towards lower levels of methylation was found in females (58.87% *vs*. 60.93, p = 0.092) and in non-mucinous tumors (59.24% *vs.* 61.41%, p = 0.096). No differences in LINE-1 methylation status were found for any of the other clinic-pathological features.

**Table 3:** LINE-1 methylation level in early-onset colorectal cancer.

Clinical, pathological or molecular features	Total (n)*	Mean	Standard deviation	p-value
Sex				
Male	61	60.93	6.598	0.092
Female	54	58.87	6.422	
Age				
>30 years	91	60.26	6.757	0.345
<30 years	24	58.83	5.791	
Body Mass Index (kg/m2)				
<30	100	59.71	6.029	0.728
>30	10	60.4	5.337	
Family history of CRC^1^				
Yes	16	59.68	6.005	0.246
No	99	61.75	9.40	
Tumor location				
Rectum	47	58.3	6.54	0.026
Distal to splenic flexure	35	60	5.46	
Proximal to splenic flexure	33	62.3	7.12	
Synchronous or metachronous CRC				
Yes	5	64.2	6.76	0.141
No	110	59.77	6.52	
TNM tumor stage				
I–II	40	59.63	6.054	0.687
III–IV	75	60.15	6.861	
Tumor differentiation				
Well or moderate	97	60.03	6.555	0.926
Poor	15	60.2	6.527	
Mucinous component				
>50%	39	61.41	5.959	0.096
<50%	75	59.24	6.824	
Medullary growth pattern				
Yes	11	61.36	4.905	0.453
No	103	59.79	6.748	
Crohńs reaction				
Yes	12	62.75	3.545	0.128
No	98	59.63	6.908	
Tumor infiltrating lymphocytes				
Yes	26	60.88	5.443	0.447
No	85	59.74	7.004	
Microsatellite instability				
MSI	25	59.72	6.717	0.454
MSS	90	60.84	6.053	
Mismatch repair deficiency^2^				
Yes	27	61.26	6.137	0.244
No	88	59.7	6.680	

P value was calculated by t-test.

* Referred to patients with available information.

1 Including first and second degree relatives.

2 MSI-H and/or loss of expression of MMR proteins by immunohistochemistry.

**Figure 1:**
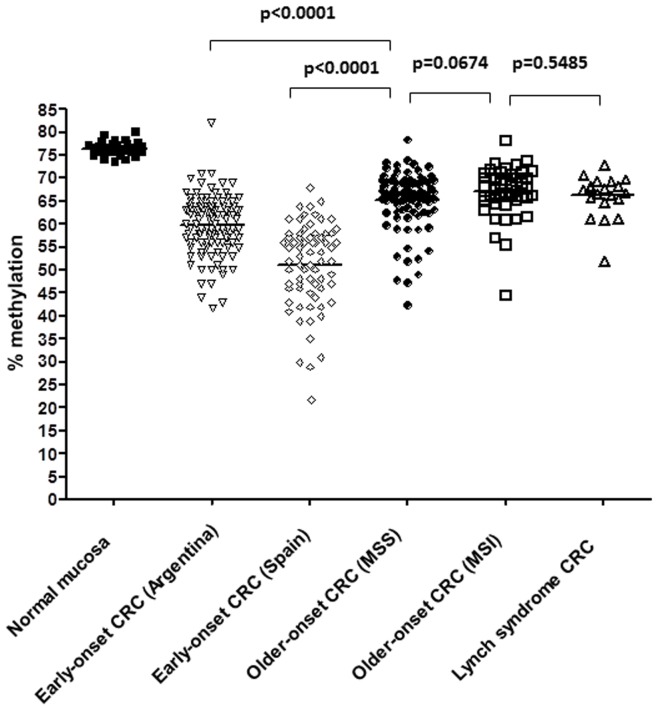
LINE-1 methylation analysis by bisulfite pyrosequencing in different CRC subsets. Bisulfite pyrosequencing of LINE-1 in colorectal tissues; Normal mucosa (n = 32), early-onset CRC from Argentina (n = 116), early-onset CRC from Spain (n = 70), older onset CRC with microsatellite stability (MSS; n = 89), older onset CRC with microsatellite instability (MSI) associated with *MLH1* promoter hypermethylation (n = 46) and Lynch syndrome CRCs (n = 20).The black horizontal bar indicates the mean methylation level.

We then compared the LINE-1 methylation levels in this series with another independent cohort of patients with CRC diagnosed at <50 years of age recruited in Spain [4], 2 groups of patients with sporadic CRC diagnosed >50 year-old categorized by the presence or absence of MSI (MSI, n = 46; mean age: 70.2+13.9 years old; and MSS, n = 89; mean age: 70.6±11.4 years old), a group of Lynch Syndrome CRCs (n = 20), and normal colonic mucosa from individuals without tumors (n = 32) (**Figure 1** and **Table 4**). Clinico-pathological features of both cohorts of early-onset CRCs are depicted in **Table S3**. Both cohorts were similar in terms of tumor location and tumor pathological features. As expected, the average LINE-1 methylation levels in normal colonic mucosa were higher than in tumor tissues for all groups. LINE-1 methylation levels in early-onset CRCs was 59.9% (SD, 6.5) and 51.1% (SD, 9.2) for the Argentinian and the Spanish cohorts, respectively. The mean methylation level in the combined cohort of early-onset CRCs (n = 185) was 56.6% (SD, 8.6). Interestingly, tumor LINE-1 methylation levels in the two independent cohorts of early-onset CRC were significantly lower than that observed in older-onset CRCs and Lynch syndrome tumors (**Table 4**), suggesting that this represents a unique feature of this subgroup of tumors (p<0.0001 for all comparisons). LINE-1 hypomethylation levels were similar in older-onset sporadic MSI tumors (67.1%, SD 5.5), Lynch syndrome CRCs (66.3%, SD 4.5), and sporadic MSS tumors (65.1%, SD 6.3).

**Table 4:** LINE-1 methylation results in different clinical subgroups.

	Mean % LINE-1 methylation (SD)	Range	p-value^1^	p-value^2^
Normal colonic mucosa (n = 32)	76.5 (1.5)	73.5–80.2		
Earlyonset CRC (n = 185)	56.6 (8.6)	22–82	<0.0001	
Lynch syndrome CRC (n = 20)	66.3 (4.5)	52.1–73.1	<0.0001	<0.0001
Older onset sporadic MSI (n = 46)	67.1 (5.5)	44.7–78.3	<0.0001	<0.0001
Older onset sporadic MSS (n = 89)	65.1 (6.3)	42.5–78.4	<0.0001	<0.0001

CRC, colorectal cancer; SD, standard deviation.

Mann Whitney test was used to compare the LINE-1 values.

1 Values for comparison between normal colonic mucosa and other groups of CRC.

2 Values for comparison between early onset CRC (n = 185) and other groups of CRC.

### Survival Analyses

Follow-up was available on all 118 patients, ranging from 1.5 to 195 months, with a mean of 39 months. The 3-year survival rate for all 118 patient in this series was 84.7%; 46 patients (39%) relapsed or had progression of disease, 22 (18.6%) died, and 3 patients were lost to follow-up. Advanced tumor stage was significantly associated with a worse 3-year overall survival (stages I-II: 92.9% vs. stages III-IV: 82.9%;p = 0.046, log rank test) and a trend was observed for better survival in patients with mucinous tumors (95.1% vs. 82.9%; p = 0.077, log rank test) or with tumor infiltrating lymphocytes (96.2% vs. 85.2%; p = 0.16, respectively; log rank test). Patients with MMR-deficient tumors showed a trend towards a better 3-year overall survival (96.3% *vs*. 83.5%; p = 0.1, log rank).

**Figure 2:**
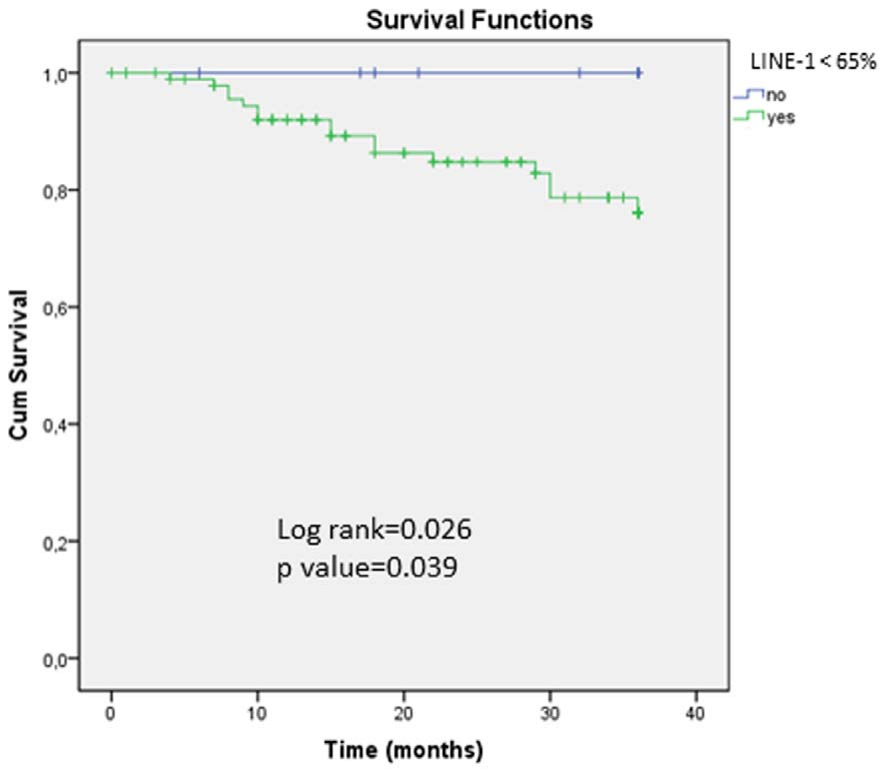
Kaplan–Meier survival curves depicting the effect of LINE-1 methylation on 3-year overall survival in early-onset CRC patients. *Vertical tick marks* indicate censored events. The green line represents survival in CRCs with LINE-1 hypomethylation <65% (n = 92) and the blue line represents LINE-1 methylation ≥65% (n = 23). p value for log rank and Fisher’s exact test are shown.

Next, we evaluated the effect of LINE-1 hypomethylation on the overall survival of CRC patients. With the aim of identifying a cut-off value of LINE-1 that could distinguish a group of patients with worse prognosis, several cut-offs starting from the lowest level of LINE-1 methylation were evaluated. We found that in comparison to patients with <65% LINE-1 methylation, those with ≥65% LINE-1 methylation had significantly better overall survival (83.5% vs. 100%; p = 0.026, log rank test; p = 0.039, Fisher’s exact test; **Figure 2**). Comparison of clinico-patological and molecular features of patients according to the level of LINE-1 methylation is shown in **Table 5**. Interestingly, tumors with LINE-1 methylation ≥65% were more frequently mucinous (60.9% vs 24.5%, p = 0.003) and those patients had higher frequency of synchronous or metachronous tumors (13% vs 2.2%, p = 0.054). Multivariate analysis showed mucinous tumors as the only variable associated with tumor with LINE-1≥65% (OR = 4.32 (95%CI: 1.65–11.34); p = 0.003), independently of the MMR deficiency status.

**Table 5:** Clinical, pathological and molecular features of patients according to the LINE-1 methylation levels.

Clinical, pathological or molecular features	LINE-1≥65% (n = 23)	LINE-1<65% (n = 92)	p-value
Sex, n (%)			
Male	9 (39.1)	45 (48.9)	0.4
Female	14 (60.9)	47 (51.1)	
Mean age (standard deviation)*	37.65 (8.3)	37.36 (8.3)	0.8
Family history of CRC^1^, n (%)			
Yes	4 (17.4)	12 (13)	0.736
No	19 (82.6)	80 (87)	
Tumor location, n (%)			
Rectum	7 (30.4)	40 (43.5)	0.209
Distal to splenic flexure	6 (26.1)	29 (31.5)	
Proximal to splenic flexure	10 (43.5)	23 (25)	
Synchronous or metachronous CRC, n (%)			
Yes	3 (13)	2 (2.2)	0.054
No	20 (87)	90 (97.)8	
TNM tumor stage, n (%)			
I–II	9 (39.5)	31 (33.7)	0.625
III–IV	14 (60.9)	61 (66.3)	
Tumor differentiation, n (%)			
Well or moderate	18 (81.8)	79 (87.8	0.489
Poor	4 (18.2)	11 (12.2)	
Mucinous component, n (%)			
>50%	14 (60.9)	25 (27.5)	0.003
<50%	9 (39.1)	66 (72.5)	
Medullary growth pattern, n (%)			
Yes	2 (8.7)	9 (9.9)	1
No	21 (91.3)	82 (90.1)	
Crohńs reaction, n (%)			
Yes	5 (21.7)	7 (8)	0.124
No	18 (78.3)	80 (92)	
Tumor infiltrating lymphocytes, n (%)			
Yes	5 (21.7)	21 (23.9)	0.83
No	18 (78.3)	67 (76.1)	
Microsatellite instability, n (%)			
MSI	6 (26.1)	19 (20.7)	0.572
MSS	17 (73.9)	73 (79.3)	
Mismatch repair deficiency^2^, n (%)			
Yes	7 (30.4)	20 (21.7)	0.379
No	16 (69.6)	72 (78.3)	

* P value was calculated by t-test.

1 Including first and second degree relatives.

2 MSI-H and/or loss of expression of MMR proteins by immunohistochemistry.

## Discussion

Several studies have suggested that early-onset CRC constitutes a biologically distinct disease that is frequently associated with advanced stage, distal tumors, and poor prognosis. We and others have shown that the known hereditary cancer syndromes only explain a minority of early-onset CRC cases [2], [4], [5], [13]; consequently, the pathogenic mechanism in the majority of cases remains unknown. In this study we aimed to gain further insight into the pathogenesis of early-onset CRC by assessing the clinic-pathological and molecular features of 118 patients with early-onset CRC. The most interesting and novel result we observed is that LINE-1 hypomethylation constitutes a unique feature of early-onset CRC patients. LINE-1 hypomethylation is a surrogate marker for genome-wide hypomethylation and is associated with increased chromosomal instability [16], [17]; therefore, this finding may help explain some of the biological mechanisms underlying early-onset CRC. In addition, we found that the frequency of MMR deficiency in this cohort is ∼20%, which is consistent with previous reports that characterized such populations [4], [5], [6]. Finally, we found that *MUTYH* deficiency accounts for ∼1% of MMR-proficient CRCs. This work and the previous work help exclude a number of possible explanations for early-onset CRC.

Cancer is a complex disease, which arises as a result of both genetic and epigenetic alterations. Human CRCs often display changes in DNA methylation, and it has been known for decades that genome-wide hypomethylation is a consistent biochemical characteristic of human colorectal tumors [16], [17], [25]. In mice, DNA hypomethylation is sufficient to induce T cell lymphomas [26]. Genome-wide hypomethylation plays a causative role in cancer through different mechanisms: genomic instability, transcriptional activation of proto-oncogenes, activation of endogenous retroviruses and transposable elements, and the induction of inflammatory mediators. All these mechanisms have been associated with DNA hypomethylation, poor prognosis and tumor aggressiveness [26], [27], [28], [29], [30], [31]. Repetitive nucleotide elements, including long interspersed nucleotide elements-1 (i.e., LINE-1) contain numerous CpG sites, and prior studies have established that the level of LINE-1 methylation is an accurate indicator of cellular 5-methylcytosine content [18], which reflects global DNA methylation. In recent years, it has been suggested that LINE-1 methylation may identify different molecular subtypes of CRC. CIMP and MSI are inversely associated with DNA hypomethylation, suggesting that genomic hypomethylation represents an alternative pathway for CRC progression, and may reflect a fundamentally different disease process [32], [33]. Moreover, LINE-1 hypomethylation has been associated with poorer survival among patients with CRC, and represents an independent factor for increased cancer-related mortality and overall mortality [20]. Therefore, evaluation of tumoral LINE-1 methylation and its correlation with clinical and pathological features is important to determine the potential clinical value of this biomarker.

We used a quantitative pyrosequencing assay for LINE-1 methylation, which is a robust, accurate and reproducible method to precisely quantitate this in individual tumors [18]. Compared to older-onset colorectal tumors, we found significantly lower levels of LINE-1 methylation in early-onset CRCs. This observation was validated in an independent set of early-onset CRC patients, reinforcing the strength of our conclusions. In addition, we found that LINE-1 hypomethylation was associated with distal tumors and worse prognosis. Although there are no previous studies that have specifically examined LINE-1 methylation in early-onset CRC patients, a recent study suggested a relationship between greater LINE-1 hypomethylation in CRC and earlier onset of the cancer (<60 years) [21]. Overall these findings suggest the presence of a distinct subtype of CRC with a unique pathogenic mechanism [4], [13]. Since the degree of LINE-1 hypomethylation is a prognostic marker in CRC and our data show that LINE-1 hypomethylation is a characteristic feature of early-onset CRC, this study provides a novel and previously unrecognized explanation for some of the biological differences (tumor location, prognosis and pathological features) involved in early-onset CRCs. These results provide an opportunity to improve our understanding of the mechanism behind early-onset CRC. In this regard, we are currently investigating whether LINE-1 hypomethylation causes direct transcriptional reactivation of certain proto-oncogenes in this setting, a unique feature that might help explain the aggressive clinical behavior of early-onset CRC. In addition, our results give rise to the hypothesis that the LINE-1 hypomethylation in peripheral blood could also be evaluated as a prognosis marker in early-onset CRCs.

Lynch syndrome is the most frequent hereditary cause of CRC, and accounts for approximately 1–3% of all CRCs [7]. It is an autosomal dominant condition caused by germline mutations in the DNA MMR genes (*MLH1, MSH2, MSH6, PMS2*), and *MSH2* and *MLH1* account for ∼90% of identifiable families. This syndrome has a gene-dependent variable penetrance for CRC and endometrial carcinoma, and an increased risk for various other extracolonic tumors. The diagnosis of Lynch syndrome has been traditionally based on tumor MMR deficiency analysis when this disease is suspected [10], [11], but the definitive diagnosis is established by finding a deleterious germline mutation in a DNA MMR gene. However, detecting Lynch syndrome is a particular challenge in the absence of a reliable family history. For this reason, universal screening with tumor MMR-deficiency analysis has been suggested [34], [35]. We have previously shown that MMR deficiency accounts for up to 20% of early-onset CRC cases [4], [5]. We also found that the pattern of MMR deficiency in early-onset CRC patients is not identical to that for all Lynch syndrome cases, and is characterized by in increased frequency of MSH6 and PMS2 deficiency. Another diagnostic challenge are MSH6-deficient CRC, as these might be missed if the screening algorithm relies entirely on MSI testing and does not include MMR immunohistochemistry [22]. In the present study, we evaluated the MMR status in an Argentinian population of early-onset CRC by analyzing both MSI and immunohistochemistry of the four MMR proteins. Twenty seven (22.9%) tumors were classified as MMR deficient. MSH2 and MLH1 deficiency accounted for the majority of cases, however, up to 20% were due to either MSH6 or PMS2 deficiency. One out of 9 MLH1-deficient cases had a *BRAF* mutation, which is typically associated with *MLH1* promoter hypermethylation. In the rest of the MLH1-deficient cases, 4 had different degrees of *MLH1* methylation, suggesting that promoter methylation might be the second hit in putative Lynch syndrome MLH1-type patients [36], [37]. It is noteworthy that 2 patients had MSI tumors with normal DNA MMR protein expression, highlighting possible limitations when using either method, since these patients would not have otherwise been identified if immunohistochemistry had been used as the only screening technique. Our results also revealed that most patients with MMR-deficient tumors did not display any significant family history of CRC or other Lynch syndrome associated tumors. These facts underscore the importance of considering the diagnosis of Lynch syndrome in all early-onset CRC even in the absence of family history, given the important clinical implications for the management of affected individuals and their relatives [38].

We found only 1 case with biallelic germline mutations in the *MUTYH* gene (p.Y176C;p.W472S) in a 29-year-old female with no family history and a MSS rectal cancer. The p.W472S variant has not been previously described and is predicted to be probably damaging based on PolyPhen 2 software analysis [39]. Therefore, novel and previously unrecognized *MUTYH* mutations should also be considered when evaluating early-onset CRC.

In spite of all the strengths, there are potential limitations of this study. First of all, comparison between groups from different geographical areas can potentially induce a selection bias. However, we were able to find a unique molecular feature in two clinical and pathologically similar cohorts of early-onset CRC from different countries, and far from a limitation, we think that this actually constitutes one of the strengths of our investigation. The differences between LINE-1 methylation in the two cohorts of early-onset CRC patients remains unexplained in this article and actually gives rise to interesting hypothesis regarding tumor biology or environmental factors influencing LINE-1 methylation. Secondly, multivariate overall survival analysis was not performed because of the limited number of patients. Larger studies are needed in the future to further confirm the association between CRC DNA hypomethylation and poorer outcomes, specifically in the early-onset patients. Also, information about chemotherapy was not available and accordingly we could not analyze the predictive value of LINE-1. On the other hand, germline MMR genetic testing was not done in patients with MMR deficiency to confirm Lynch syndrome; for MLH1-deficient cases, we ruled out CIMP-associated tumors by analyzing the V600E *BRAF* mutation and *MLH1* methylation status. Since somatic inactivation of *MSH2*, *MSH6*, and *PMS2* are rare events, we hypothesized that most patients with MMR deficiency in this study are putative Lynch syndrome cases. Finally, we only studied the 2 most prevalent MUTYH mutations in Caucasian population, which account for >85% of cases. Despite the fact that in Argentina >75% of the population are European descendants, our results may be underestimated due to the presence of mutations other than G393D or Y176C.

In summary, we have studied a large cohort of early-onset CRC cases and found that LINE-1 hypomethylation in these tumors constitutes a unique and specific feature compared with older-onset CRC, which is suggestive of a distinct molecular subtype of these colorectal neoplasms. Our results suggest that the LINE-1 methylation status could be used as prognostic biomarker for young people with CRC. Future studies are needed to confirm this association and to understand the mechanisms by which DNA hypomethylation affects CRC prognosis, as well as to examine whether this feature could have therapeutic implications.

## Supporting Information

Figure S1 (PPTX)

Table S1 **Pyrosequencing primer description.**
(DOCX)

Table S2 **Clinicopathological and molecular features of** ***MUTYH*** **mutation carriers.**
(DOCX)

Table S3 **Clinical, pathological and molecular features of early-onset CRC recruited in Spain and Argentina.**
(DOC)
