# Supplementary figures and images for: A High Degree of LINE-1 Hypomethylation Is a Unique Feature of Early-Onset Colorectal Cancer
Source: PLoS One. 2012 Sep 25;7(9):e45357. doi: 10.1371/journal.pone.0045357 (PMC3458035; doi:10.1371/journal.pone.0045357)

## Slide 1
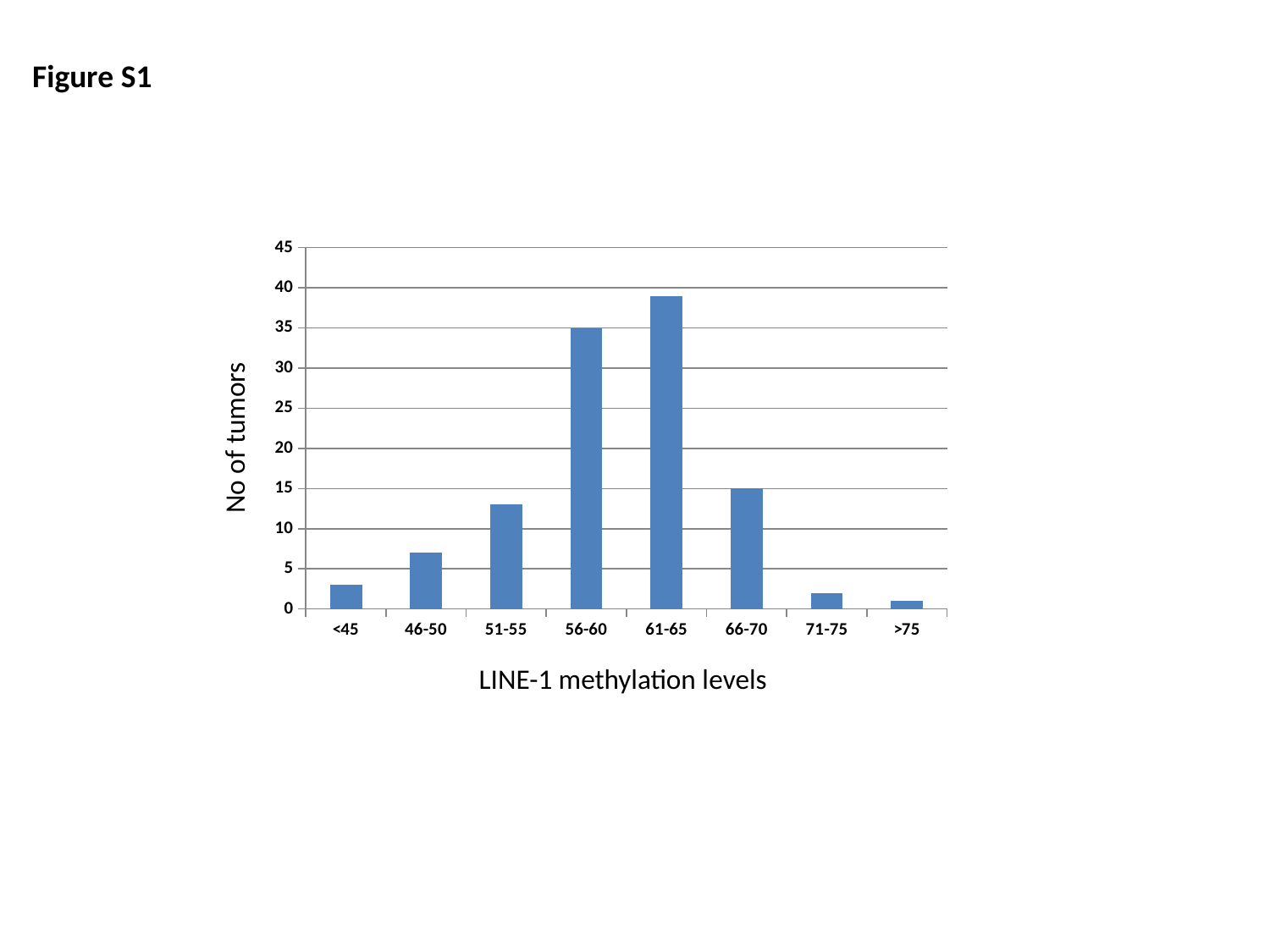

Figure S1
### Chart
| Category | |
|---|---|
| <45 | 3.0 |
| 46-50 | 7.0 |
| 51-55 | 13.0 |
| 56-60 | 35.0 |
| 61-65 | 39.0 |
| 66-70 | 15.0 |
| 71-75 | 2.0 |
| >75 | 1.0 |No of tumors
LINE-1 methylation levels

Supplement: Figure S1 — (PPTX) [file pone.0045357.s001.pptx]
