# Supplementary material for: A High Degree of LINE-1 Hypomethylation Is a Unique Feature of Early-Onset Colorectal Cancer
Source: PLoS One. 2012 Sep 25;7(9):e45357. doi: 10.1371/journal.pone.0045357 (PMC3458035; doi:10.1371/journal.pone.0045357)
Supplement: Table S1 — Pyrosequencing primer description. (DOCX) [file pone.0045357.s002.docx]

| **Assay** | **Forward** | **Reverse (5’biotinilated)** | **Sequencing** | **Product size** | **Sequence to analyze** |
| --- | --- | --- | --- | --- | --- |
| MUTYH Y176C | ACACAGGAGGTGAATCAACTCTG | CCAAGACTCCTGGGTTCCTAC | GGAGGTGAATCAACTCTG | 118 | GGCTGGCCTGGGCTA/GCTA TTCTCGTGGCCGGCGGCTGCAG |
| MUTYH  G393D | GGCTGCCCTCCCTCTCA | AGGTCACGGACGGGAACTC | TGCCCTCCCTCTCAG | 57 | G/ATCTGCTGGCAGGACTGTGGGAGTTC |
| BRAF | GAAGACCTCACAGTAAAAATAG | ATAGCCTCAATTCTTACCATCC | AGGTGATTTTGGTCTAGCTACAG | 122 | A/TGAAATCT |
| LINE-1 | TTTTGAGTTAGGTGTGGGATATA | AAAATCAAAAAATTCCCTTTC | AGTTAGGTGTGGGATATAGT | 150 | TT**YG**TGGTG**YG**T**YG**TTTTTTAAGT**YG**GTTTGAAAAG**YG**T |
| MLH1 | GAAATTTGATTGGTATTTAAGTTGTTTAAT | TCAACCAATCACCTCAATACCTC | TGATTGGTATTTAAGTTGTTT | 119 | AATTAATAGTTGT**YG**TTGAAGGGTGGGGTTGGATGG**YG**TAAGTTATAGTTGAAGGAAGAA**YG**TGAGTA**YG**AGG |

**Table S1: Pyrosequencing primer description**

Y indicates C/T
