# Supplementary material for: A High Degree of LINE-1 Hypomethylation Is a Unique Feature of Early-Onset Colorectal Cancer
Source: PLoS One. 2012 Sep 25;7(9):e45357. doi: 10.1371/journal.pone.0045357 (PMC3458035; doi:10.1371/journal.pone.0045357)
Supplement: Table S2 — Clinicopathological and molecular features of MUTYH mutation carriers. (DOCX) [file pone.0045357.s003.docx]

**Table S2: Clinicopathological and molecular features of *MUTYH* mutation carriers**

| Case | Age | Sex | Tumor location | Stage | Differentiation | Mucinous production | MSI status | MMR IHC | Other tumors | Synchronous adenomas (number) | MUTYH mutation |
| --- | --- | --- | --- | --- | --- | --- | --- | --- | --- | --- | --- |
|  |  |  |  |  |  |  |  |  |  |  |  |
| 020ARG | 42 | Male | Splenic flexure | IV | Moderately | No | MSS | Normal | No | No | G382D/- |
| 064ARG | 39 | Male | Cecum | IIB | Moderately | Yes | MSS | Normal | Rectum (41) | Yes (3) | G382D/- |
| 074ARG | 29 | Female | Rectum | IIIA | Moderately | No | MSS | Normal | No | Yes (2) | Y176C/W472S |
