# Supplementary material for: A High Degree of LINE-1 Hypomethylation Is a Unique Feature of Early-Onset Colorectal Cancer
Source: PLoS One. 2012 Sep 25;7(9):e45357. doi: 10.1371/journal.pone.0045357 (PMC3458035; doi:10.1371/journal.pone.0045357)
Supplement: Table S3 — Clinical, pathological and molecular features of early-onset CRC recruited in Spain and Argentina. (DOC) [file pone.0045357.s004.doc]

**Table S3: Clinical, pathological and molecular features of early-onset CRC recruited in Spain and Argentina.**

| **Clinical, pathological or molecular features** | **Early-onset CRC from Spain**  **(n=70)** | **Early-onset CRC from Argentina**  **(n=118)** | **p value** |
| --- | --- | --- | --- |
| Sex, n (%) |  |  |  |
| Female | 32 (45.7) | 61 (51.7) | 0.454 |
| Male | 38 (54.3) | 57 (48.3) |  |
| Mean age at diagnosis (standard deviation)* | 43.81 (6.6) | 37 (8.25) | 0.0001 |
| Family history of CRC^1^, n (%) |  |  |  |
| Yes | 24 (35.3) | 15 (12.7) | 0.0001 |
| No | 43 (63.2) | 103 (87.3) |  |
| Tumor location, n (%) |  |  |  |
| Rectum | 28 (40) | 49 (41.5) | 0.5 |
| Distal to splenic flexure | 26 (37.1) | 35 (29.7) |  |
| Proximal to splenic flexure | 16 (22.9) | 34 (28.8) |  |
| Synchronous or metachronous CRC, n (%) |  |  |  |
| Yes | 2 (2.9) | 6 (5.1) | 0.712 |
| No | 68 (97.1) | 112 (94.9) |  |
| TNM tumor stage, n (%) |  |  |  |
| I-II | 29 (42.6) | 41 (34.7) | 0.284 |
| III-IV | 39 (57.4) | 77 (65.3) |  |
| Tumor differentiation, n (%) |  |  |  |
| Well or moderate | 62 (88.6) | 100 (86.9) | 0.821 |
| Poor | 8 (11.4) | 15 (13.1) |  |
| Mucinous component, n (%) |  |  |  |
| >50% | 18 (25.7) | 41 (34.7) | 0.197 |
| <50% | 52 (74.3) | 77 (65.3) |  |
| Medullary growth pattern, n (%) |  |  |  |
| Yes | - | 11 (9.4) | - |
| No | - | 106 (90.6) |  |
| Crohn´s reaction, n (%) |  |  |  |
| Yes | - | 12 (10.6) | - |
| No | - | 101 (89.4) |  |
| Tumor infiltrating lymphocytes, n (%) |  |  |  |
| Yes | 4 (6) | 26 (22.8) | 0.003 |
| No | 63 (94) | 88 (77.2) |  |
| Mismatch repair deficiency^2^, n (%) |  |  |  |
| Yes | 10 (14.3) | 27 (22.9) | 0.186 |
| No | 60 (85.7) | 91 (77.1) |  |

*P value was calculated by t-test

^1^ Including first and second degree relatives

^2^ MSI-H and/or loss of expression of MMR proteins by immunohistochemistry
